# Global trends and current status in colistin resistance research: a bibliometric analysis (1973-2019)

**DOI:** 10.12688/f1000research.25124.1

**Published:** 2020-07-31

**Authors:** Abdourahamane Yacouba, Ahmed Olowo-okere

**Affiliations:** 1 Faculté des Sciences de la Santé, Université Abdou Moumouni, Niamey, Niger; 2 Faculty of Pharmaceutical Sciences, Usmanu Danfodiyo University, Sokoto, Nigeria

**Keywords:** colistin resistance, mcr-1, keywords, bibliometric analysis

## Abstract

**Background**: Colistin resistance is a major breach in our last line of defense and without urgent action, we are heading for a post-antibiotic era, in which common infections and minor injuries can once again kill. To the best of our knowledge, the use of the bibliometric analytical technique for examining colistin resistance-related research does not exist in the literature.

**Methods**: Here, we analyze and present bibliometric indicators of the global literature in colistin resistance research. The Scopus database was searched for articles on colistin resistance. The articles retrieved were analyzed using the bibliometrix R-package.

**Results**: A total of 1105 publications were retrieved. There was a noticeable increase in the number of publications on colistin resistance research in the past decade. Six journals made up the core zone in colistin research and produced 35.83% of the published articles. The analysis across time-intervals revealed several keywords that had increased or decreased in usage when comparing the interval between 1973-2009 and 2010-2019. Authors’ keywords *“Acinetobacter baumanii”*, and “ *Pseudomonas aeruginosa”* were the most frequent encountered during the period of 1973-2009, while “ *mcr-1*”, “ *Enterobacteriaceae*”, “ *Escherichia coli*”, and “ *Klebsiella pneumoniae*” emerged in the past decade.

**Conclusions**: There has been a significant growth in publications on colistin resistance in the past decade, suggesting an urgent need for action by different stakeholders to contain this threat of colistin resistance. Keyword analysis revealed temporal changes in the types of keywords used across time-intervals. These findings summarize a general vision on colistin resistance research and will serve as baseline data for future comparative purposes.

## Introduction

According to the World Health Organization (WHO), antibiotic resistance is one of today's greatest threats to global health, food security, and development ^1^. The emergence and unprecedented spread of carbapenemases have led to a resurgence in the use of last-line antibiotic colistin as salvage therapy ^2– 5^.

Colistin or polymyxin E is a polypeptide antibiotic developed in the 1950s. Originally, the antibiotic was isolated from the soil bacterium *Paenibacillus polymyxa subsp* ^6^. Though withdrawn from clinical use in the 1980s owing to its significant toxicity, it has been re-introduced for the treatment of multidrug resistant (MDR) bacteria, particularly carbapenem-resistant Gram-negative bacterial infections ^4, 5, 7, 8^. It acts by interfering with bacterial cell membranes and/or the inhibition of bacterial respiration ^9, 10^.

In 2016, the first plasmid-mediated colistin resistance gene, *mcr-1*, was detected in *Escherichia coli and Klebsiella pneumoniae* ^11^. Since then, several variants of *mcr-1* and novel families of *mcr* genes have been identified in a variety of samples ^12– 18^. Colistin resistance is a major breach in our last line of defense and without urgent action, we are heading for a post-antibiotic era, in which common infections and minor injuries can once again kill.

Bibliometrics is the branch of library science that applies mathematical and statistical techniques to analyze articles, reviews, editorial letters, books, and other documents ^19^. This is an important tool to assess new trends in scientific research. To the best of our knowledge, the use of the bibliometric analytical technique for examining colistin resistance-related research does not exist in the literature. Here, we analyze and present bibliometric indicators of the global literature in colistin resistance research.

## Methods

### Literature search

Articles on colistin resistance published in the literature were retrieved from the Scopus database on November 15, 2019. Keywords used for data extraction were obtained from published review articles on colistin resistance ^20– 22^. The search query used for data extraction was: TITLE-ABS-KEY ("colistin resistance" OR "polymyxin E resistance").

### Study selection

Two authors independently performed the literature search using EndNote X9 (Bld 12062) produced by Clarivate Analytics. Papers published between January 1, 1973 and 31 December 2019 were included. After removal of duplicate results, titles and abstracts were reviewed for relevance to the research question. Any disagreement was resolved by consensus of the two authors. Experimental studies and studies that reported colistin sensitive bacteria were not eligible for inclusion. Searches were restricted to studies published in the English language.

### Analysis methods

According to similar previously published bibliometric studies, selected documents were analyzed by different standard indicators including total number of publications, document types, countries/territories, authors, sources, total number of citations, h-index, average number of citations per publication, international collaborations and Bradford division analysis using the bibliometrix R-package ^23, 24^. Furthermore, graphs were plotted for visualization of co-citation networks and keyword co-occurrence of the text corpus extracted from the title and the abstract fields of the articles ^25^. Co-citation has been defined as any two items (authors) that have been jointly cited by another item (author). To determine temporal changes in the type of keywords in colistin resistance research, analysis was carried out for two-time intervals of the study period: period 1, representing 1973 to 2009, and period 2, from 2010 to 2019. All data analysis was performed using the open-source bibliometrix R-package ^26^ on RStudio version 1.2.1335 (Boston, MA). The bibliometrix R-package provides a set of functions for quantitative research in bibliometric, including: (1) data loading and conversion to R dataframe (readFiles(), convert2df()), (2) data analysis (summary() and plot(), citations(), local citations(), Hindex(), lotka(), keywordGrowth(), keywordAssociation()), and (3) data visualization (biblioNetwork(), histNetwork()) ^26^.

## Results

### Publications outputs and trends

**Figure 1.**
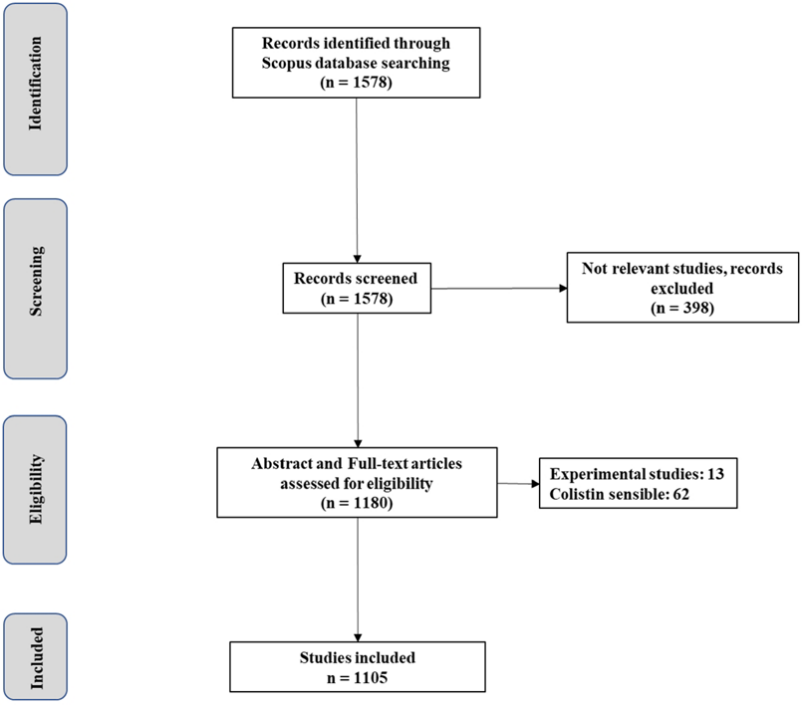
Flow diagram of the selection process of the included studies.

A total of 1578 non duplicate publications indexed in Scopus were retrieved on November 15, 2019. Of the 1578 studies, 1105 met the inclusion criteria and were used for the bibliometric analysis ( Figure 1) ^27^. The result of distribution by time indicates that the literature in this field was first published in 1973. A significant increase occurred in the number of publications from 35 in the period 1973-2009 to 1070 in the past decade (P value <0.001). Most of the publications were original research studies ( *n* = 862; 78.01%), followed by editorial letters ( *n* = 124; 11.22%), and reviews ( *n* = 65; 5.88%).

### Sources analysis

The top 10 cited papers, their citation frequency and titles are listed in Table 1. The most-cited article ( *n* = 1632) was written by Liu *et al*. (2016), followed by papers authored by Olaitan *et al.* (2014) ( *n* = 412) and Moffatt *et al.* (2010) ( *n* = 336). The top 10 preferred sources for publishing documents on colistin resistance are shown in Table 2. Bradford division analysis shows six sources in the core zone. These six sources are *Antimicrobial Agents and Chemotherapy*, *Journal of Antimicrobial Chemotherapy*, *International Journal of Antimicrobial Agents, Frontiers in Microbiology*, *Journal of Global Antimicrobial Resistance*, and *The Lancet Infectious Diseases*, which produced 35.83% of the published articles.

**Table 1.** The top 10 cited papers.

Top ten most cited original articles						
	Authors	Year	Source	Title	TC	TCperYear ^a^
1	Liu *et al*. ^11^	2016	Lancet Infect Dis	Emergence of plasmid-mediated colistin resistance mechanism MCR-1 in animals and human beings in China: a microbiological and molecular biological study	1632	544.00
2	Moffatt *et al*. ^28^	2010	Antimicrob Agents Chemother	Colistin resistance in Acinetobacter baumannii is mediated by complete loss of lipopolysaccharide production	336	37.30
3	Adams *et al*. ^29^	2009	Antimicrob Agents Chemother	Resistance to colistin in Acinetobacter baumannii associated with mutations in the PmrAB two-component system	247	24.70
4	Tumbarello *et al*. ^30^	2015	J Antimicrob Chemother	Infections caused by KPC-producing Klebsiella pneumoniae: differences in therapy and mortality in a multicentre study	232	58.00
5	Yin *et al*. ^15^	2017	MBio	Novel plasmid-mediated colistin resistance gene mcr-3 in Escherichia coli	204	102.00
6	Hasman *et al*. ^31^	2015	Eurosurveillance	Detection of mcr-1 encoding plasmid-mediated colistin- resistant Escherichia coli isolates from human bloodstream infection and imported chicken meat, Denmark 2015	199	49.80
7	Beceiro *et al.* ^32^	2011	Antimicrob Agents Chemother	Phosphoethanolamine modification of lipid A in colistin- resistant variants of Acinetobacter baumannii mediated by the pmrAB two-component regulatory system	195	24.40
8	Carattoli *et al*. ^33^	2017	Eurosurveillance	Novel plasmid-mediated colistin resistance mcr-4 gene in Salmonella and Escherichia coli, Italy 2013, Spain and Belgium, 2015 to 2016	161	80.50
9	Capone *et al.* ^34^	2013	Clin Microbiol Infect	High rate of colistin resistance among patients with carbapenem-resistant Klebsiella pneumoniae infection accounts for an excess of mortality	160	26.70
10	Hussein *et al.* ^35^	2009	Infect Control Hosp Epidemiol	Impact of carbapenem resistance on the outcome of patients' hospital-acquired bacteraemia caused by Klebsiella pneumoniae	160	16.00
Top ten most cited review articles						
1	Olaitan *et al*. ^36^	2014	Frontiers in Microbiology	Mechanisms of polymyxin resistance: Acquired and intrinsic resistance in bacteria	412	58.85
2	Cai *et al.* ^37^	2012	J Antimicrob Chemother	Colistin resistance of Acinetobacter baumannii: clinical reports, mechanisms and antimicrobial strategies	284	40.57
3	Poirel *et al*. ^6^	2017	Clinical Microbiology Reviews	Polymyxins: Antibacterial activity, susceptibility testing, and resistance mechanisms encoded by plasmids or chromosomes	241	34.42
4	Lim *et al.* ^38^	2010	Pharmacotherapy	Resurgence of colistin: a review of resistance, toxicity, pharmacodynamics, and dosing	182	20.22
5	Yahav *et al*. ^39^	2012	Clin Microbiol Infect	Colistin: new lessons on an old antibiotic	136	19.43
6	Barbier *et al*. ^40^	2013	Curr Opin Pulm Med	Hospital-acquired pneumonia and ventilator-associated pneumonia: recent advances in epidemiology and management	111	18.50
7	Ruppe E *et al*. ^41^	2015	Ann Intensive Care	Mechanisms of antimicrobial resistance in Gram-negative bacilli	101	25.25
8	Bialvaei *et al.* ^42^	2015	Curr Med Res Opin	Colistin, mechanisms and prevalence of resistance	91	22.75
9	Catry *et al.* ^43^	2015	Int J Antimicrob Agents	Use of colistin-containing products within the European Union and European Economic Area (EU/EEA): development of resistance in animals and possible impact on human and animal health	69	17.25
10	Sun *et al.* ^44^	2018	Trends Microbiol	Towards understanding MCR-like colistin resistance	65	65.00

^a^By the end of year 2018; TC, total citation.

**Table 2.** The top 10 sources that published articles in colistin resistance research.

Rank	Sources	Articles	%
1	Antimicrobial Agents And Chemotherapy	126	44.37%
2	Journal Of Antimicrobial Chemotherapy	79	27.82%
3	International Journal Of Antimicrobial Agents	73	25.70%
4	Frontiers In Microbiology	44	15.49%
5	Journal Of Global Antimicrobial Resistance	40	14.08%
6	Lancet Infectious Diseases	34	11.97%
7	Microbial Drug Resistance	29	10.21%
8	Infection And Drug Resistance	23	8.10%
9	Eurosurveillance	22	7.75%
10	Clinical Microbiology And Infection	20	7.04%

### Countries/regions analysis

**Figure 2.**
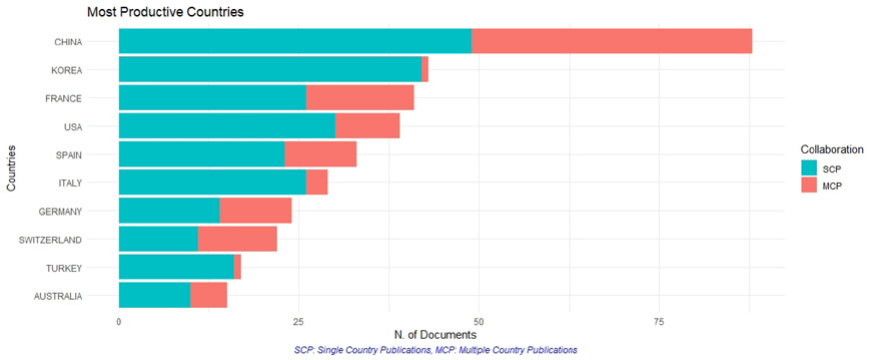
Top 10 countries with the most publications

**Figure 3.**
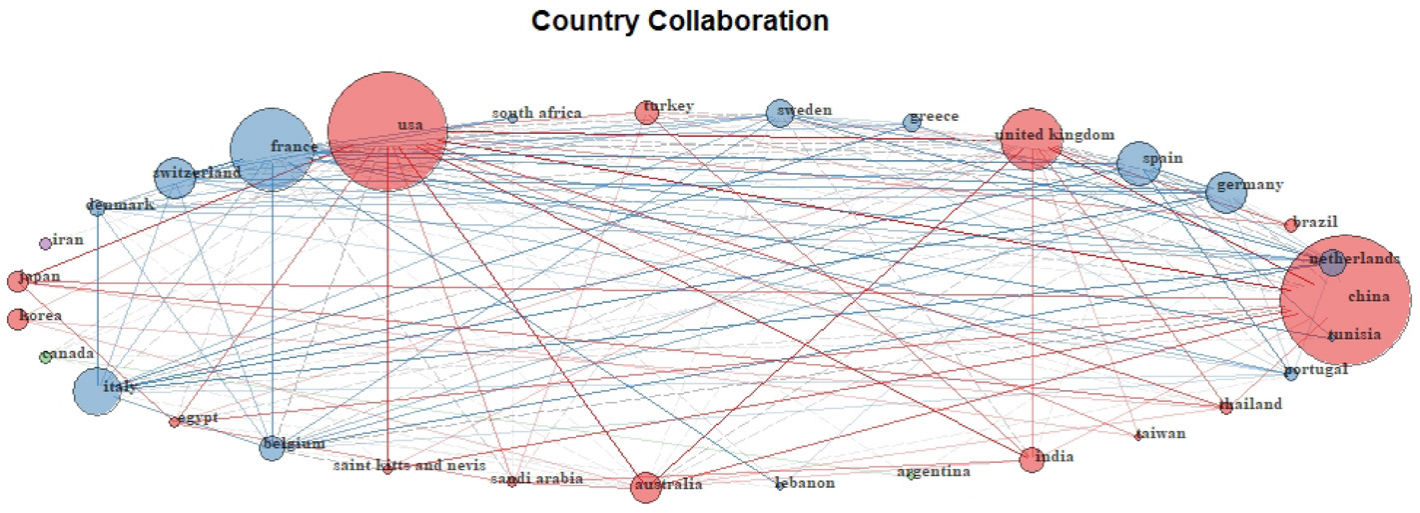
International collaboration analysis for top 30 active countries. Each node in the figure symbolizes a different country’ collaboration with other countries. The node’s diameter corresponds to the strength of collaboration. Links represent international collaboration pathways between countries. Cluster of countries having similar cluster color most probably represents a closely related research group. Links represent the strength of collaboration.

The top 10 most productive territories included two Asian countries, seven European countries and one country in North America. China dominated the literature in colistin resistance research with 212 items (19.18%), followed distantly by the USA ( *n* = 166; 15.02%) and France ( *n* = 120; 10.85%). China also had the highest citation frequency ( Figure 2). International collaboration analysis for active countries is shown in Figure 3.

### Author analysis

**Figure 4.**
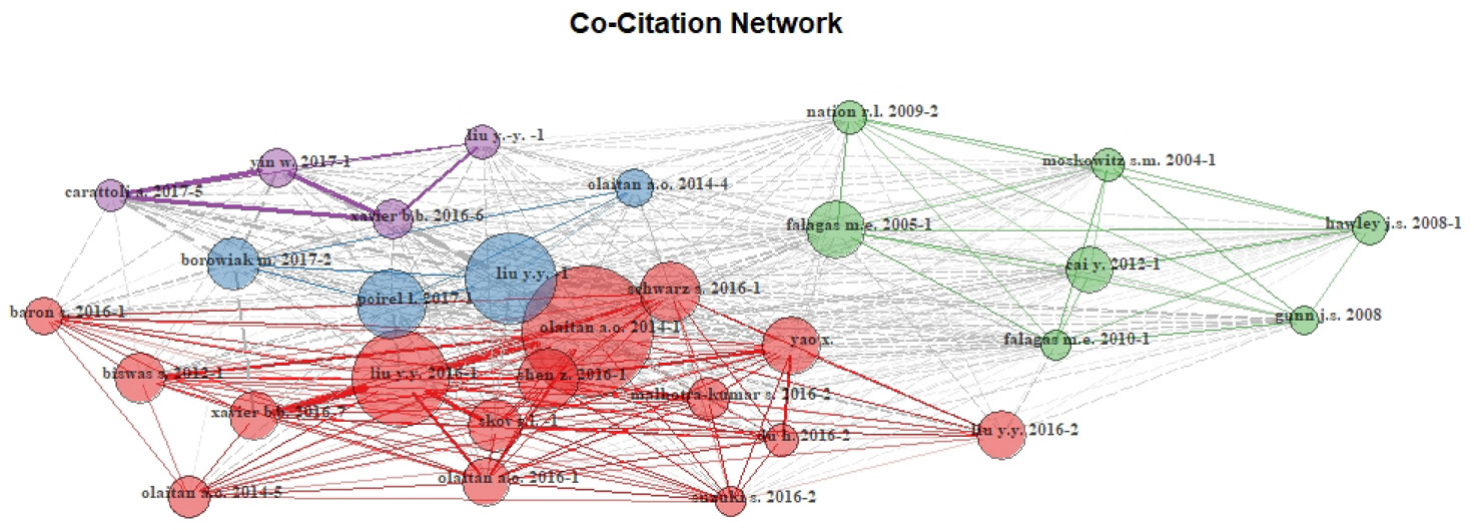
The co-citation network map of references from publications in colistin resistance research Each node in the network symbolizes a different author’ co-citation. The node’s diameter corresponds to the strength of co-citation. Links represent co-citation pathways between authors. Co-citation network of authors showed four clusters. Links diameter represent the strength of co-citation.

The 1105 publications were written by 5141 authors working on this subject. Author analysis highlighted 0.215 documents per author, 4.65 authors per document, and 7.37 co-authors per document, with a collaboration index of 4.74. A co-citation network of authors showed four clusters: the blue cluster dominated by Liu 2016; the red cluster dominated by Olaitan 2014; the green cluster dominated by Falagas 2005 and the purple cluster dominated by Xavier 2016 ( Figure 4).

### Keywords analysis

Temporal changes in the type of keywords were observed between the period 1 (1973 to 2009) and period 2 (2010 to 2019) ( Table 3).

**Table 3.** Types of keywords observed in period 1 (1973-2009) and period 2 (2010-2019).

Period 2 (2010 – 2019)				
	Author keywords (DE)	Articles	Keywords- Plus (ID)	Articles
1	Colistin resistance	232	Colistin	1834
2	Colistin	213	Antibiotic resistance	895
3	*mcr*-1	196	*Escherichia coli*	802
4	*Escherichia coli*	85	Nonhuman	769
5	*Klebsiella pneumoniae*	80	Bacterial	756
6	*Acinetobacter baumannii*	63	Article	689
7	Antibiotic resistance	59	*Klebsiella pneumoniae*	598
8	Antimicrobial resistance	52	Human	597
9	*Enterobacteriaceae*	51	Drug resistance	583
10	Resistance	45	Anti bacterial agents	527
Period 1 (1973 – 2009)				
1	Colistin	12	Colistin	56
2	*Acinetobacter baumannii*	5	Article	28
3	Cystic fibrosis	4	Antibiotic resistance	26
4	Resistance	4	Drug resistance	26
5	Colistin resistance	3	*Acinetobacter baumannii*	24
6	Polymyxin	3	Bacterial	22
7	*Pseudomonas aeruginosa*	3	Nonhuman	21
8	*Acinetobacter*	2	Anti bacterial agents	19
9	Antibiotic resistance	2	*Pseudomonas aeruginosa*	18
10	Etest	2	Humans	16

## Discussion

The aim of this study was to analyze the global scientific outputs of colistin resistance research and show the trends and current status in colistin resistance research. The publication trend showed a significant increase in the number of publications, particularly in the past decade, from 2010 to 2019, which corresponds with the peak clinical interest in its use as antibiotic of choice against MDR Gram-negative bacteria. This trend reflects the increasing attention paid by different stakeholders to concerns about colistin resistance worldwide and shows that research aiming to reduce the threat of colistin resistance is gaining attention in the scientific community.

Bradford division analysis showed a high concentration (35.83%) of publications in a few journals, namely *Antimicrobial Agents and Chemotherapy*, *Journal of Antimicrobial Chemotherapy, International Journal of Antimicrobial Agents*, *Frontiers in Microbiology*, *Journal of Global Antimicrobial Resistance,* and *The Lancet Infectious Diseases*. Four of these six sources are exclusively dedicated to antimicrobials, which is consistent with the global effort against antimicrobial resistance.

China was the leading country for publications related to colistin resistance, contributing 19.18% of all Scopus database articles published during the period of study. This may be due to the widespread use of colistin in animal feedstock and agriculture in China ^45^. Moreover, the fact that China was the first country where a plasmid-mediated mechanism of colistin resistance, the mobilizable colistin resistance gene-1 ( *mcr-1*), was reported, may be a contributing factor to China’s commitment ^11^. The majority of publications on colistin resistance research were produced by high-income countries, with a negligible contribution from low- and middle-income countries, particularly Africa, no countries in which were on the list of the top ten most productive countries. Several previous bibliometric studies of the literature have also reported a similar distribution of research publications in different fields from high-income countries ^23, 46, 47^. This poor research output from African countries could be due to the lack of funding and poor facilities.

With regards to keywords, the analysis across time-intervals revealed that several keywords have increased or decreased in usage when comparing the interval between 1973-2009 and 2010-2019. This shows a temporal change in the type of keywords. Keywords “ *mcr-1*”, “ *Enterobacteriaceae”*, “ *Escherichia coli”*, and “ *Klebsiella pneumoniae”*, which were absent from 1973 to 2009, have emerged in the past decade. This could be attributed to the fact that until the first report of *mcr-1* gene, colistin resistance in *Enterobacteriaceae* was believed to be chromosomally mediated ^36^. However, since 2016, the *mcr-1* gene was reported in *Enterobacteriaceae* recovered from food, animals, and human specimens in China, and it has subsequently been reported worldwide ^11, 48– 53^. The wide spread of the *mcr-1* dramatically challenges the newly renewed interest in colistin for clinical use and opens a new research topic on colistin. Currently, eight new *mcr* genotypes ( *mcr*-2 to *mcr*-9) have been reported since the discovery of *mcr-1* ^14– 18, 33, 54, 55^. Furthermore, colistin-resistant bacteria without prior colistin exposure, possibly due to cross-resistance between colistin and cationic antimicrobials such as LL-37 and lysozyme, have also been reported ^56, 57^.

Unsurprisingly, the article “ *Emergence of plasmid-mediated colistin resistance mechanism mcr-1 in animals and human beings in China: a microbiological and molecular biological study*,” published by the Lancet ^11^ was the most cited article.

Country collaboration in colistin resistance research remains relatively weak. This raises the fundamental question of global collaboration to slow the development and spread of colistin resistance ^58^. Country collaboration may be the most practical solution to this transmissible plasmid-mediated resistance in the era of globalization, with increased migration, trade and travel. Indeed, horizontal transfer of *mcr*-mediated colistin resistance is a rapid phenomenon, and can not only occur at a rather high frequency but can also disseminate across different bacterial species. Following its first description, plasmid- *mcr* has now been reported across all seven continents ^59^.

### Strengths and limitations of research

To the best of our knowledge, this study is the first to initiate baseline data on bibliometrics in colistin resistance research. The study was not limited by language and a large literature including original articles, reviews, editorial letters and meeting abstracts published over a long period was included. The Scopus database covers the vast majority of online research, particularly broader biomedical research, and it has been established that it is the most user-friendly and easiest tool to use for bibliometric analysis services ^60, 61^. Furthermore, comprehensive and relatively objective data analysis was performed, which clearly highlighted the past and current status of colistin resistance research and predicted the future research frontier.

However, this global study is characterized by a number of limitations that are inherent in bibliometric analysis ^62^. The main limitation of the present data is the reliance solely upon the Scopus database, which did not represent all the literature. Despite the fact that the Scopus database is considered an excellent source for bibliometric analysis, there are some journals that contain publications on colistin resistance but are not indexed in Scopus and therefore were not counted. Furthermore, we included papers where colistin resistance or polymyxin E resistance was used in the title or abstract or keywords; that inclusion of search items gives a much lower sensitivity to the search.

## Conclusions

There has been a significant growth of publications on colistin resistance in the past decade, suggesting an urgent need for action by different stakeholders to contain this threat of colistin resistance. Unsurprisingly, an article on *mcr-1* gene research ranked first among the top ten most cited articles on colistin resistance. Keyword analysis revealed temporal changes in the types of keywords used across time-intervals. These findings summarize a general vision on colistin resistance research and will serve as baseline data for future comparative purposes.

## Data availability

### Underlying data

Figshare: Full list of included studies .bib. https://doi.org/10.6084/m9.figshare.12673151.v1 ^27^.

Data are available under the terms of the Creative Commons Zero "No rights reserved" data waiver (CC0 1.0 Public domain dedication). ****
