# Supplementary material for: Global trends and current status in colistin resistance research: a bibliometric analysis (1973-2019)
Source: F1000Res. 2020 Jul 31;9:856. [Version 1] doi: 10.12688/f1000research.25124.1 (PMC7786423; doi:10.12688/f1000research.25124.1)
Supplement: Peer review history

# Peer review: Reviewer response for version 1

**DOI:** 10.5256/f1000research.27727.r74440

**Published:** 2021-01-05

**Authors:** Ismaeel Yunusa

**Affiliations:** 1 Department of Clinical Pharmacy and Outcomes Sciences, University of South Carolina, College of Pharmacy, Columbia, SC, USA

The authors used the bibliometric analytical technique to examine research related to colistin resistance. In the absence of any previous study on the current state of research for this problem, I find this study timely. However, I have the following comments for improvement: 

1. Under the "Strengths and limitations of research" section, authors mentioned that "the study was not limited by language"; however, in the "Methods" Section, they mentioned that "Searches were restricted to studies published in the English language.". This means that a focus on English language articles actually limited the study. Authors may need to modify as appropriate. 
2. Authors may need to mention how they handled articles with full-texts in another language, but abstract and other author characteristics are in English. 
3. Authors may provide further context on a one-sentence paragraph in the discussion. Or consider incorporating the sentence into another paragraph, if appropriate. 
4. Presenting a graph, if possible, that illustrates the trend in publications over time might be helpful. The title itself reads global trends, so a graph showing temporal relationships is necessary. 

Is the work clearly and accurately presented and does it cite the current literature?

Yes

If applicable, is the statistical analysis and its interpretation appropriate?

Yes

Are all the source data underlying the results available to ensure full reproducibility?

Yes

Is the study design appropriate and is the work technically sound?

Yes

Are the conclusions drawn adequately supported by the results?

Yes

Are sufficient details of methods and analysis provided to allow replication by others?

Yes

Reviewer Expertise:

Research methodologist. Internal Medicine, Oncology, Geriatrics, and Infectious diseases

I confirm that I have read this submission and believe that I have an appropriate level of expertise to confirm that it is of an acceptable scientific standard.

# Peer review: Reviewer response for version 1

**DOI:** 10.5256/f1000research.27727.r70235

**Published:** 2020-09-07

**Authors:** David Lupande-Mwenebitu

**Affiliations:** 1 Hôpital Provincial Général de Référence de Bukavu, Université Catholique de Bukavu (UCB), Bukavu, Democratic Republic of the Congo; 2 Center for Tropical Diseases and Global Health, Catholic University of Bukavu, Bukavu, Democratic Republic of the Congo

The work is interesting, with this merit indeed of having used bibliometrics as a tool, however, several other databases must be consulted including Google Scholar, Web of Science and Pubmed to come out as large as possible. To work over a long period and to find only less than 2000 published papers seems insufficient to me.

 Currently, there are 10 types of mcr gene, mcr-10 was described last March ^1^.

Is the work clearly and accurately presented and does it cite the current literature?

Yes

If applicable, is the statistical analysis and its interpretation appropriate?

Yes

Are all the source data underlying the results available to ensure full reproducibility?

Yes

Is the study design appropriate and is the work technically sound?

Yes

Are the conclusions drawn adequately supported by the results?

Yes

Are sufficient details of methods and analysis provided to allow replication by others?

Partly

Reviewer Expertise:

Clinical Microbiologist, Antibiotics resistance, Hygien and noscomial infections surveillance

I confirm that I have read this submission and believe that I have an appropriate level of expertise to confirm that it is of an acceptable scientific standard.

## References

[1] : Identification of novel mobile colistin resistance gene mcr-10. *Emerg Microbes Infect*.2020;9(1) : 10.1080/22221751.2020.1732231 508-516 10.1080/22221751.2020.1732231 32116151PMC7067168
